# Accumulation of Histidine Reduce the Susceptibility of *Vibrio splendidus* Persister Cells to Tetracycline

**DOI:** 10.1111/1758-2229.70383

**Published:** 2026-06-30

**Authors:** Yanan Li, Xinnan Pan, Jiayao Chen, Hong Ding, Yina Shao

**Affiliations:** ^1^ School of Marine and Biological Engineering, Yancheng Teachers University Yancheng Jiangsu China; ^2^ GuXin Bioengineering Equipment (Jiangsu) Co. Ltd Yancheng Jiangsu China; ^3^ State Key Laboratory for Managing Biotic and Chemical Threats to the Quality and Safety of Agro‐Products, Ningbo University Ningbo Zhejiang China

**Keywords:** histidine metabolism, membrane potential, persister cells, tetracycline, *Vibrio splendidus*

## Abstract

*Vibrio splendidus* is an opportunistic pathogen widely distributed in marine environments, animal tissues and seabed sediments. It can infect various marine animals, resulting in high mortality and substantial economic losses. Exposure to high concentrations of tetracycline induces *V. splendidus* to form persister cells, which exhibit tolerance to multiple classes of antibiotics and pose a serious threat to aquaculture by promoting disease outbreaks. Previous studies have suggested that metabolic regulation is a key mechanism for maintaining the dormant and low‐energy state of persister cells. In this study, we found that the accumulation of histidine‐derived metabolites significantly reduced the susceptibility of *V. splendidus* to tetracycline. Transcriptomic analysis revealed a marked upregulation of genes involved in histidine metabolism and exogenous supplementation of histidine significantly increased the proportion of persister cells, suggesting that histidine plays a promotive role in persister formation. Furthermore, histidine enhanced the membrane potential and upregulated the expression of the efflux pump gene *tolC*, thereby contributing to the formation of tetracycline‐induced persister cells. These findings reveal a previously unrecognized role of histidine metabolism in antibiotic tolerance and provide a theoretical basis for understanding persister cell formation in *V. splendidus* .

## Graphical abstract

Histidine accumulation promotes tetracycline‐induced persister formation in *V. splendidus* . It enhances membrane potential and upregulating the efflux pump gene, thereby reducing antibiotic susceptibility.

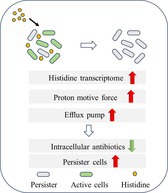

## 1 Introduction

*Vibrio splendidus* is a Gram‐negative, rod‐shaped bacterium of the genus Vibrio that is highly pathogenic, causing skin ulcer syndrome (SUS) in *Apostichopus japonicus* and widely distributed in seawater, sediments and the tissues or organs of various marine organisms (Thompson et al. 2004; Farmer 2006; Takemura et al. 2014; Zhang and Li 2021). Prophylactic or therapeutic use of antibiotics in aquaculture can impose selection pressure on natural bacterial populations, potentially promoting the development of antibiotic tolerance in these environments (Hossain et al. 2022). Moreover, established antibiotic tolerance poses persistent and intractable threats to both aquatic ecosystems and human health (Hossain et al. 2020). Notably, within bacterial populations, there exists a small dormant subpopulation capable of tolerating the bactericidal effects of high concentrations of antibiotics—persister cells (Wiuff et al. 2005). A previous study revealed that a small fraction of the *V. splendidus* population can survive exposure to tetracycline at 10 times the MIC. When exposed to lethal antibiotic concentrations, a small subset of persister cells inevitably survives and exhibits reversible, non‐genetic tolerance to multiple drugs (Wiuff et al. 2005; Li et al. 2021). Persister cells do not acquire genetic mutations; once the antibiotic pressure is removed, they resume normal growth and regain drug susceptibility. Such phenotypic persistence is widespread among bacterial pathogens and contributes to both the development of antibiotic tolerance and the frequent relapse of chronic infections in humans and in aquaculture (Helaine and Kugelberg 2014). It has been reported that tetracycline can induce the formation of persister cells in *V. splendidus* (Li et al. 2021). Although tetracycline has broad‐spectrum antibacterial activity and low toxicity, it remains a widely used antibiotic for disease prevention in aquaculture (Hu et al. 2022).

The inducing factors and formation mechanisms of persister cells are complex and diverse. Nutrient deprivation, environmental changes (e.g., shifts in pH, temperature, or osmotic pressure), oxidative stress, extracellular signal molecules and antimicrobial agents can all induce the formation of persister cells (Fisher et al. 2017). Bacteria activate multiple pathways to respond to these triggers, thereby leading to the formation of persister cells. For example, bacteria employ mechanisms such as reducing ATP levels, increasing the formation of protein aggregates, inhibiting DNA replication or transcription, arresting translation and decreasing intracellular antibiotic concentrations to form persister cells (Van den Bergh et al. 2017). Recent studies have explored the mechanisms of persister cell formation via bacterial metabolic self‐regulation. Lim et al. established a hypoxia‐induced persister cell model of *Mycobacterium tuberculosis* and then used isotope‐resolved metabolomics to characterize metabolic changes in these cells. They found that phosphoenolpyruvate, a key intermediate in central carbon metabolism, was almost completely depleted in persisters; this depletion further restricted carbon flux into pathways essential for bacterial replication and antibiotic sensitivity. Conversely, supplementation with phosphoenolpyruvate restored carbon fluxes, which promoted *M. tuberculosis* growth and enhanced its drug susceptibility, ultimately preventing the emergence of tolerance (Lim et al. 2021; Derewacz et al. 2013). Metabolomic analysis of *Nocardiopsis* persister cells revealed that *Nocardiopsis* increased tetrahydropyrimidine levels by modulating fatty acid metabolism, thereby contributing to its drug tolerance (Derewacz et al. 2013). In addition, studies have shown that when *Edwardsiella tarda* is cultured in a medium supplemented with glucose or alanine, metabolic flux analysis revealed a redirection of carbon flow toward the glyoxylate cycle, which has been demonstrated to play a regulatory role in bacterial drug tolerance (Su et al. 2018). Rydzak's research team adopted metabolic preference analysis as a diagnostic strategy, which used metabolite consumption and excretion patterns from in vitro microbial cultures to identify pathogens and assess their antimicrobial susceptibility (Rydzak et al. 2022). Therefore, investigating how bacterial metabolic regulation influences the drug tolerance of persister cells provides a crucial theoretical foundation for their prevention and treatment.

Histidine is an essential amino acid required for protein synthesis and also acts as a signalling molecule involved in host immune regulation (Ning et al. 2024). Studies have shown that histidine metabolism is a key metabolic pathway for maintaining the survival of bacteria in a persistent state. *M. tuberculosis* resists host histidine starvation by activating the de novo histidine biosynthesis pathway, while the histidine‐deficient strain *ΔhisD* fails to proliferate within macrophages and the bacterial load in the lungs of infected mice is significantly reduced (Dwivedy et al. 2021).

Bacteria need to maintain energy homeostasis through metabolic reprogramming in the persister state and the activation of the histidine synthesis pathway ensures bacterial survival in nutrient deficient environments (Hinshelwood and Stoker 1992). The histidinol phosphate phosphatase HolPase‐homologous proteins PA0335 and PA3255 in *Pseudomonas aeruginosa* not only catalyse histidine biosynthesis but also affect bacterial tolerance to ciprofloxacin and meropenem by regulating biofilm formation (Guo et al. 2025). In addition, *Staphylococcus aureus* *vraS* mutations activate histidine biosynthesis genes and increase peptidoglycan cross‐linking in the cell wall, thereby reducing the binding sites for β‐lactam antibiotics (Fang et al. 2025). However, how histidine metabolism regulates persister cell formation and tolerance in *V. splendidus* remains unknown.

In this study, we conducted a global transcriptomic analysis of *V. splendidus* , both tetracycline‐induced persister cells and active cells. By examining gene expression profiles across three distinct bacterial growth stages, we found that genes involved in histidine biosynthesis and degradation were significantly upregulated in *V. splendidus* persister cells. To further explore this mechanism, an exogenous histidine supplement and 3‐amino‐1,2,4‐triazole (3‐AT) were used to modulate intracellular histidine levels. Notably, the accumulation of histidine and histidine‐related metabolites reduced intracellular tetracycline concentrations and enhanced the tolerance of the aquaculture pathogen *V. splendidus* to tetracycline. These findings suggest that histidine metabolism may play a key role in the formation or maintenance of the persister state, providing new insights into the metabolic regulation underlying bacterial drug tolerance.

## 2 Materials and Methods

### 2.1 Bacterial Strain and Culture Conditions

The *V. splendidus* strain used in this study was originally isolated from a diseased *A. japonicus* collected at a hatchery in Jinzhou and preserved in our laboratory. This strain has been deposited in the China General Microbiological Culture Collection (CGMCC, Beijing, China) under accession number 7.242 (Jiang et al. 2022). The bacterial culture was incubated in 2216E medium (2216E) at 28°C with shaking at 180 rpm. The medium consisted of 5 g/L yeast extract and 0.01 g/L FePO_4_, prepared with filtered natural seawater. The antimicrobial agents tetracycline, ciprofloxacin, ampicillin and kanamycin were purchased from Shanghai Sangon Biological Engineering Technology & Services Co. Ltd. (Shanghai, China). L‐histidine and 3‐AT were purchased from Beijing Solarbio Life Sciences Co. Ltd. (Beijing, China) and were of analytical grade.

### 2.2 Measurement of Minimal Inhibitory Concentration

The minimal inhibit concentration (MIC) was determined following the protocol described by Zhang et al. (Dwivedy et al. 2021). Briefly, an overnight culture of *V. splendidus* was diluted 1:100 (vol/vol) and inoculated into fresh 2216E medium, then incubated at 28°C until the optical density at 600 nm (OD_600_) reached 0.5. Then, 10 μL of bacterial suspension (1.0 × 10^5^ CFU/mL) was added to each well of a 96‐well polystyrene microplate, along with 100 μL of two‐fold serial dilutions of the respective tested antibiotic. The plates were incubated at 28°C for 16 h. The MIC was defined as the lowest concentration that completely inhibited visible bacterial growth. All assays were performed in triplicate and repeated independently three times.

### 2.3 Antibiotic‐Induced Persister Cells

Persister cells were induced and isolated as previously described by Lewis et al. (Kwan et al. 2013; Lewis 2007). An overnight culture of *V. splendidus* was inoculated into 50 mL of 2216E broth and incubated at 28°C with shaking at 180 rpm for 16 h. Specifically, tetracycline was added to a final concentration of 250 μg/mL (10 × MIC), and the culture was incubated at 28°C for 4 h to eliminate non‐persister cells. Then, 1 mL aliquots of the culture were harvested, washed and serially diluted in PBS. A 10 μL aliquot of each dilution were spot‐plated onto 2216E agar plates. Colony‐forming units were enumerated after 16 h incubation at 28°C.

### 2.4 Transcriptome Analysis

Sample preparation was performed as previously described by Keren et al. (Keren et al. 2011), with slight modifications. An overnight culture of *V. splendidus* was diluted 1:100 (vol/vol) in fresh 2216E broth and incubated at 28°C with shaking at 180 rpm. Bacterial cells were collected at three growth phases (OD_600_ = 0.6, 1.2, 2.0) by centrifugation at 6000 × g for 5 min. The resulting pellets were resuspended in 2216E broth containing 250 μg/mL tetracycline and incubated at 28°C for 4 h to eliminate non‐persister cells. Cells were then washed three times with PBS and centrifuged under the same conditions.

The Ribo‐zero Kit was used to remove rRNA to enrich mRNA. Subsequently, the mRNA was cleaved into short fragments with a lysis reagent. First‐strand cDNA was synthesized using random hexamer primers and mRNA as templates. Buffer, DNA polymerase I, RNase H and dNTPs were added to synthesize double‐stranded cDNA. The resulting cDNA was purified with AMPure XP beads to obtain the final library. Sequencing was performed on the Illumina HiSeq/MiSeq sequencing platform operated by Novogene Biotechnology (Beijing, China). Illumina original reads were pruned and quality controlled to obtain clean reads. Clean reads were mapped to the genome using Bowtie2 (Langmead and Salzberg 2012).

### 2.5 Analysis of Differential Gene Expression

Total RNA was isolated from *V. splendidus* cells using TRIzol reagent (Invitrogen, Thermo Fisher Scientific, Waltham, MA, USA) according to the manufacturer's instructions. qPCR was performed in 20 μL reaction volumes using an Applied Biosystems (ABI) 7500 Real‐Time PCR System, with each reaction containing SYBR Premix Ex Taq (TaKaRa, Dalian, China) and diluted cDNA. The thermal cycling program consisted of initial denaturation at 95°C for 5 min, followed by 40 cycles of denaturation at 95°C for 15 s, annealing at 60°C for 20 s and extension at 70°C for 20 s, with fluorescence detection at the end of each extension step. A melting curve was generated by ramping the temperature to 95°C at a rate of 0.1°C/s to confirm the specificity of PCR amplicons. Relative gene expression levels were calculated using the 2^−ΔΔCt^ quantification method.

### 2.6 Measurement of Intracellular Histidine Concentration

Intracellular histidine concentration was quantified with the Histidine Enzyme‐Linked Immunosorbent Assay (ELISA) Kit (Longchangshuo, Cat. No. MK530155A, Shanghai, China) according to the manufacturer's instructions. Bacterial cells were harvested by centrifugation at 5000 ×*g* for 5 min and washed three times with PBS. Five millilitres of the cell suspension was then sonicated in an ice bath with the following parameters: 30% output power, 2 s sonication, 8 s interval, for a total duration of 2 min. The cell lysate supernatant was collected and used for histidine quantification according to the ELISA kit instructions.

Absorbance values were measured at 450 nm using a microplate reader (Molecular Devices, SpectraMax 190, Sunnyvale, CA, USA). A standard curve was generated by plotting known histidine concentrations versus their respective optical density (OD_450_) values. Sample histidine concentrations were calculated using the standard curve equation. Intracellular histidine concentration was determined by normalizing the measured histidine concentrations to cell number and single‐cell volume.

### 2.7 Statistical Analysis

Data are presented as the mean ± standard deviation (SD). One‐way analysis of variance (ANOVA) was used to assess statistical significance, with *p* < 0.05, *p* < 0.01 and *p* < 0.001 considered statistically significant. Statistical analyses were performed using OriginPro 2022 (OriginLab, Northampton, MA, USA).

## 3 Results

### 3.1 Characterization and Growth Phase Dependence of *V. splendidus* Persister Cells

**FIGURE 1:**
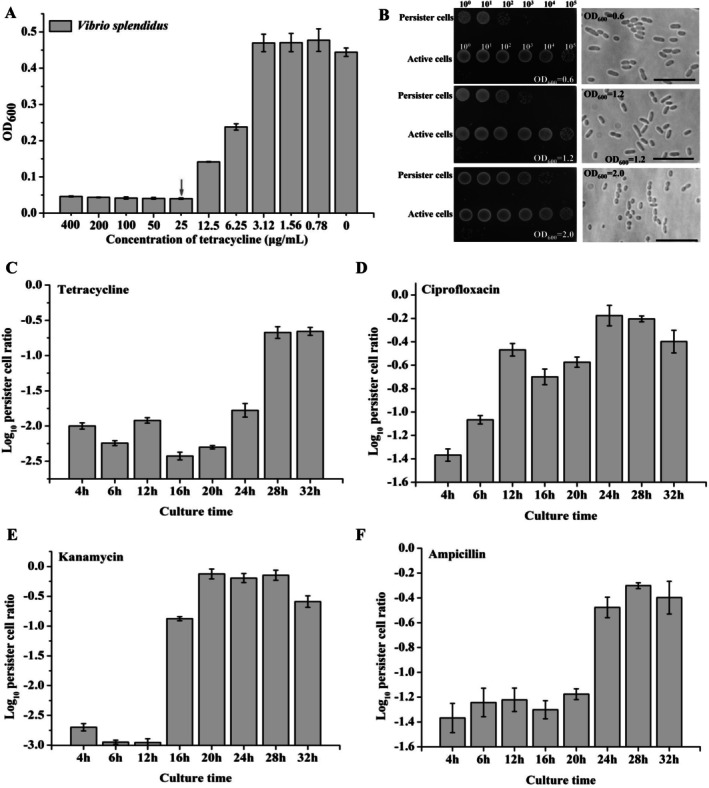
Minimal inhibitory concentration of tetracycline and quantification of antibiotic survival. (A) The arrow indicates the concentration of tetracycline in 2216E medium that prevented the growth of *V. splendidus* . (B) Early exponential phase, mid‐exponential phase and stationary phase of *V. splendidus* exposed to antibiotics at 10 × MIC. Ten‐fold serial dilutions of *V. splendidus* were grown to overnight and spotted onto 2216E agar plates. Bright‐field images of cells showing the morphology of active cells and tetracycline‐induced persister cells at different stages of growth (scale bar, 10 μm). (C‐F) Growth stage dependence of persister formation in *V. splendidus* . Frequency of persister cells formation as function of culture time from inoculation, determined by antibiotic susceptibility measurement (antibiotic including: 250 μg/mL tetracycline, 125 μg/mL ciprofloxacin, 250 μg/mL kanamycin and 125 μg/mL ampicillin for 4 h at 28°C, respectively).

Using the serial dilution method to determine the MIC (Zhang et al. 2019), we found that the MIC of tetracycline against *V. splendidus* was 25 μg/mL (Figure 1A). In our previous study, we defined *V. splendidus* persister cells as those that survived 10 × MIC tetracycline treatment for 4 h (Li et al. 2021), a definition consistent with the methodology described by Keren et al. (Kwan et al. 2013; Lewis 2007; Keren et al. 2011). Subsequently, we observed the number and single‐cell morphology of tetracycline‐induced *V. splendidus* persister cells at three growth phases. The number of persister cells increased as the bacterial culture time increased, which might be attributed to nutrient depletion in the medium. In addition, single cells exhibited a long rod shape at OD_600_ values of 0.6 and 1.2, but a small coccoid shape at OD_600_ = 2.0 (Figure 1B). We used different antibiotics (ciprofloxacin, kanamycin, ampicillin) to induce persister cells and determined the persister formation rate of bacteria at different culture time points. Consistent with the above observations, the persister formation rate also increased as the bacterial culture duration increased (Figure 1C–F).

### 3.2 Histidine Metabolism Enriched in Transcriptomic GO Analysis

**FIGURE 2:**
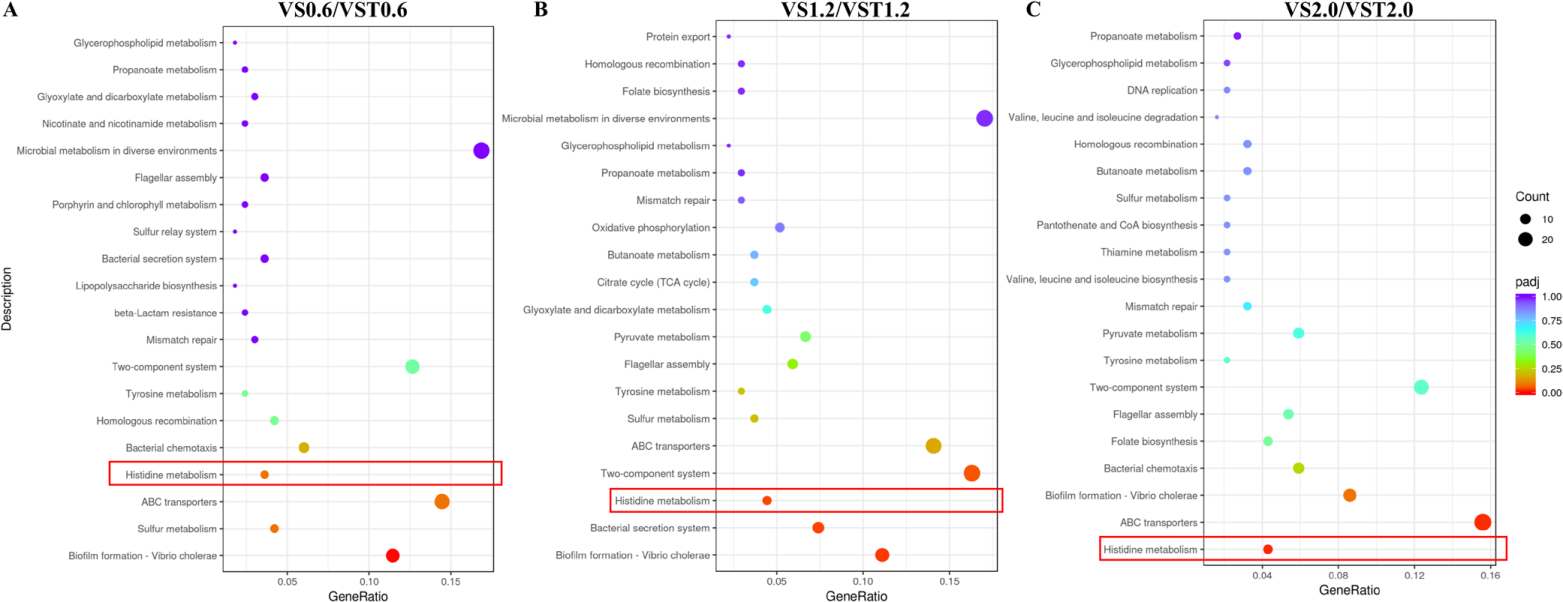
Histidine metabolism enriched in transcriptomic GO analysis. (A) Top 20 significantly enriched KEGG pathways among differentially expressed genes in tetracycline‐induced *V. splendidus* persister cells at OD_600_ = 0.6. (B) Top 20 significantly enriched KEGG pathways among differentially expressed genes in tetracycline‐induced *V. splendidus* persister cells at OD_600_ = 1.2. (C) Top 20 significantly enriched KEGG pathways among differentially expressed genes in tetracycline‐induced *V. splendidus* persister cells at OD_600_ = 2.0. VS0.6/VST0.6, VS1.2/VST1.2 and VS2.0/VST2.0 (VS: active cells; VST: tetracycline‐induced persister cells) represent transcriptomic comparisons between persister cells and active cells when the bacterial culture reached an OD_600_ of 0.6, 1.2 and 2.0, respectively. The red arrow indicates the histidine metabolism pathway.

**FIGURE 3:**
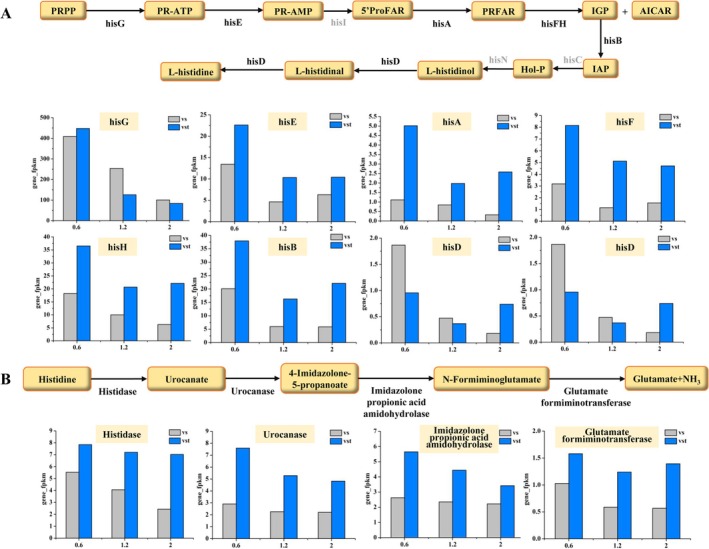
Histidine metabolism is upregulated in tetracycline‐induced *V. splendidus* persister cells. (A) Simplified diagram of the histidine biosynthetic pathway in *V. splendidus* persister cells. Enzyme abbreviations are shown in bold; grey font indicates unannotated genes; corresponding metabolites are shown in yellow boxes; FPKM values of genes with differential expression between VST0.6, VST1.2, VST2.0 and their respective active cell controls (VS0.6, VS1.2, VS2.0) are illustrated by column graphs. (B) Simplified diagram of the histidine catabolism pathway in *V. splendidus* persister cells. Enzyme abbreviations are shown in bold; grey font indicates unannotated genes; corresponding metabolites are shown in yellow boxes; FPKM values of genes with differential expression between VST0.6, VST1.2, VST2.0 and their respective active cell controls (VS0.6, VS1.2, VS2.0) are illustrated by column graphs.

Among the differentially upregulated metabolic pathways in tetracycline‐induced persister cells, histidine metabolism was notably enriched. Histidine metabolism is one of the important metabolic pathways in bacterial energy metabolism (Akashi and Gojobori 2002). Transcriptomic comparisons between VS0.6/VST0.6, VS1.2/VST1.2 and VS2.0/VST2.0 (VS: active *V. splendidus* cells; VST: tetracycline‐induced *V. splendidus* persister cells) revealed significant upregulation of the histidine metabolism pathway, particularly in VST2.0 (Figure 2A–C). The biosynthesis of L‐histidine begins with the critical metabolite phosphoribosyl pyrophosphate. The enzymes responsible for histidine metabolism were encoded by the his operon, which includes the genes *hisG*, *hisE*, *hisI*, *hisA*, *hisF*, *hisH*, *hisB*, *hisC*, *hisN* and *hisD* (Brosnan and Brosnan 2020). The transcriptomic FPKM data showed increased expression of most his operon genes in tetracycline‐induced persister cells, except for *hisG* and *hisD*, which were downregulated (Figure 3A). Additionally, genes involved in the histidine catabolism pathway play a key role in the formation of tetracycline‐induced *V. splendidus* persister cells, potentially contributing to the survival of these persister cells under antibiotic stress (Figure 3B). These results indicate that histidine metabolism may be closely involved in the formation of tetracycline‐induced *V. splendidus* persister cells.

### 3.3 Histidine Accumulation Enhances Persister Cells

**FIGURE 4:**
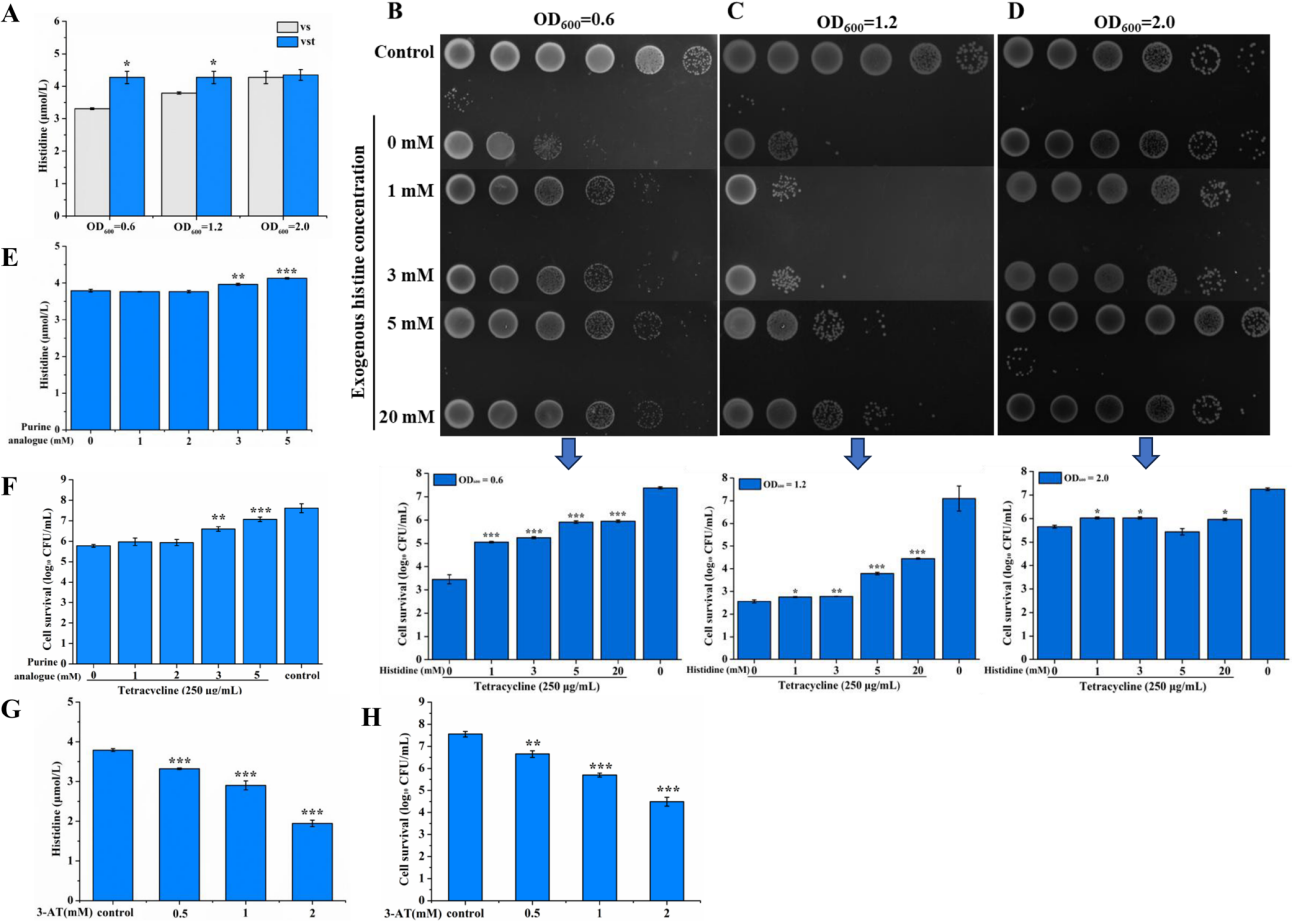
Intracellular histidine accumulation enhances tetracycline persistence in *V. splendidus* persister cells. (A) Intracellular histidine concentrations between tetracycline‐induced *V. splendidus* persister cells and active cells at different growth phases. (B–D) Effects of exogenous histidine on tetracycline tolerance of *V. splendidus* at different growth phases, early exponential phase (OD_600_ = 0.6), mid‐exponential phase (OD_600_ = 1.2) and stationary phase (OD_600_ = 2.0). Cultures were supplemented with exogenous histidine at the following concentrations: 0 1, 3, 5 and 20 mM. (E) Intracellular histidine concentrations in *V. splendidus* after exogenous purine analogue treatment. (F) Persister cell survival rate of *V. splendidus* after exogenous purine analogue treatment, assessed via tetracycline tolerance assay. (G) Intracellular histidine concentrations in *V. splendidus* after treatment with 3‐AT, an inhibitor of histidine biosynthesis. (H) Persister cell survival rate of *V. splendidus* after treatment with 3‐AT, assessed via tetracycline tolerance assay. The bars indicated the mean of at least three independent experiments; error bar indicate STDEV. (*p < 0.05; **p < 0.01; ***p < 0.001).

To determine whether histidine contributes to the formation of tetracycline‐induced *V. splendidus* persister cells, we performed the following experiments. First, the bacterial counts were normalised; we quantified intracellular histidine concentrations in *V. splendidus* at different growth phases. A standard curve was established by plotting known histidine concentrations against their corresponding optical density (OD_450_) values. The analysis revealed statistically higher intracellular histidine levels in tetracycline‐induced *V. splendidus* persister cells compared with active cells at OD_600_ = 0.6 (*p* < 0.05) and OD_600_ = 1.2 (*p* < 0.05); however, no significant difference was observed at OD_600_ = 2.0 (*p* > 0.05) (Figure 4A). To further assess the functional role of histidine metabolites in modulating drug susceptibility, we supplemented exogenous histidine during tetracycline‐induced *V. splendidus* persister cell formation. As expected, histidine supplementation enhanced *V. splendidus* persister cell survival in a dose‐dependent manner across all growth phases. At OD_600_ = 0.6, 1, 3, 5 and 20 mM histidine led to 36‐fold (*p* < 0.001), 55‐fold (*p* < 0.001), 267‐fold (*p* < 0.001) and 289‐fold (*p* < 0.001) increases in persister cell survival, respectively, relative to the control group without histidine supplementation (Figure 4B). At OD_600_ = 1.2, persister cell survival was 0.55‐fold (*p* < 0.05), 0.65‐fold (*p* < 0.01), 15‐fold (*p* < 0.001) and 76‐fold (*p* < 0.001) that of the control, respectively (Figure 4C), while at OD_600_ = 2.0, the corresponding survival rates were 1.2‐fold (*p* < 0.05), 1.2‐fold (*p* < 0.05), 0.28‐fold (*p* > 0.05) and 1.1‐fold (*p* < 0.05) that of the control (Figure 4D).

These findings indicate that intracellular histidine accumulation may play a protective role against tetracycline stress, particularly during the early and mid‐exponential phases, potentially contributing to *V. splendidus* persister cell formation. Histidine metabolism and purine metabolism are subject to reciprocal negative feedback regulation (Moffatt and Ashihara 2002). Therefore, the addition of purine analogues (6‐mercaptopurine) led to a dose‐dependent increase in intracellular histidine concentration in *V. splendidus* , which in turn resulted in a corresponding increase in the persister cell formation rate (Figure 4E,F). Although this regulatory relationship has been well characterized in fungal species such as *Aspergillus fumigatus* , the enzyme IGPD catalyses the sixth step in histidine biosynthesis and is the first pathway‐specific enzyme in this process (Busch et al. 2001; Dietl et al. 2016). To validate the importance of histidine biosynthesis in *V. splendidus* persister cell formation, we used 3‐AT, a competitive inhibitor of IGPD, to suppress histidine synthesis (Dietl et al. 2016; Ahangar et al. 2013). As expected, treatment with increasing concentrations of 3‐AT reduced intracellular histidine concentrations in *V. splendidus* (Figure 4G), concomitantly resulting in a dose‐dependent decrease in the survival rate of tetracycline‐induced *V. splendidus* persister cells (Figure 4H). These results collectively indicated that histidine accumulation significantly enhances persister cell formation in *V. splendidus* through protecting cells against tetracycline stress.

### 3.4 Increased Proton Motive Force Stimulates Antibiotic Efflux in the Presence of Histidine

**FIGURE 5:**
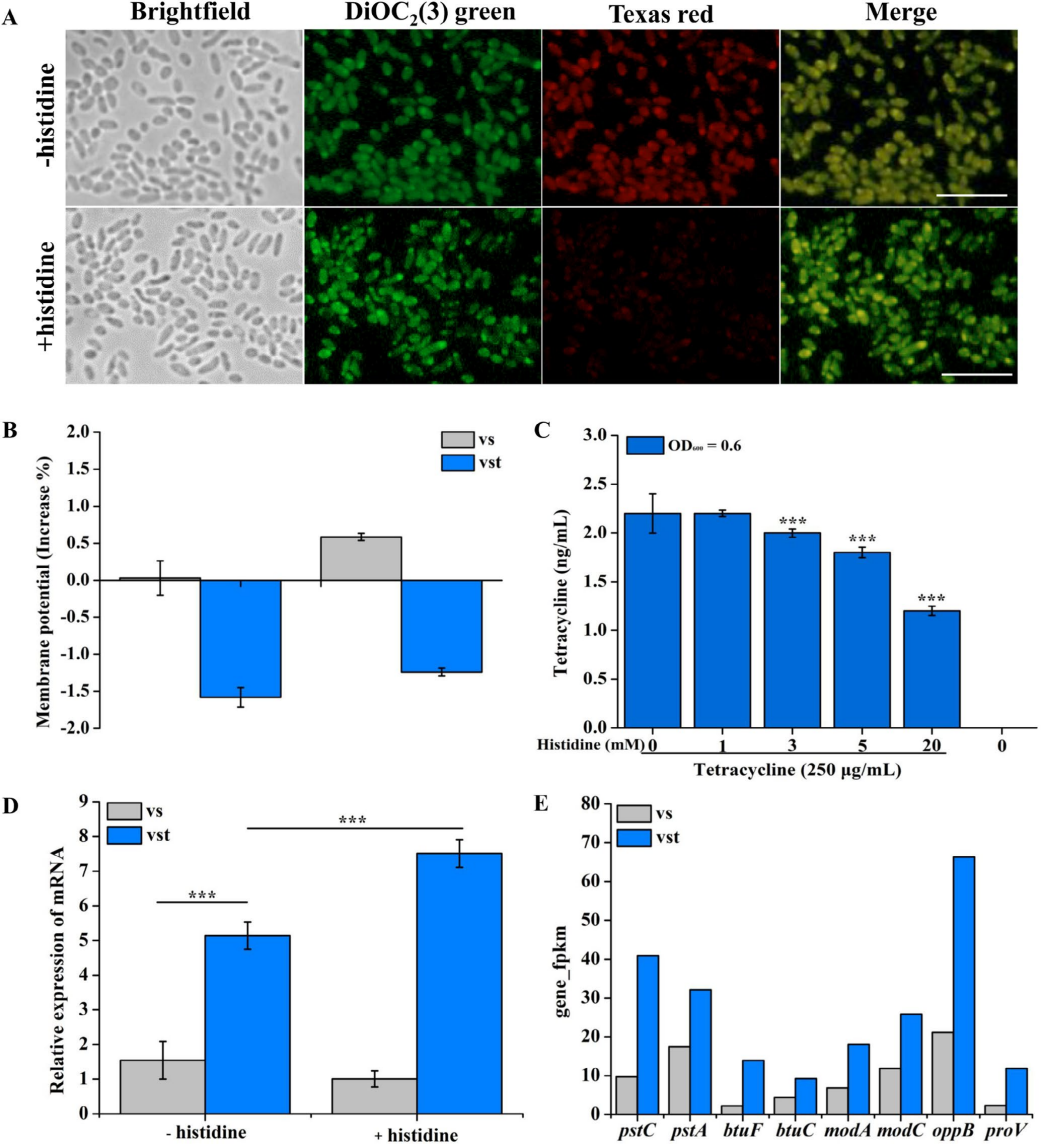
Histidine accumulation affects membrane potential and intracellular tetracycline concentration in *V. splendidus* . (A) Membrane potential fluorescence images of *V. splendidus* in the presence or absence of exogenous histidine. The scale bar is 10 μm. Microscopy settings were consistent across all images. (B) Relative changes in membrane potential of *V. splendidus* in the presence or absence of exogenous histidine. (C) Intracellular tetracycline concentrations in *V. splendidus* in the presence or absence of exogenous histidine. (D) Expression of the efflux pump gene *tolC* in *V. splendidus* in the presence or absence of exogenous histidine. (E) FPKM values of ABC transporter‐related genes in *V. splendidus* persister cells. The bars indicated the mean of at least three independent experiments; error bar indicated STDEV. (*p < 0.05; **p < 0.01; ***p < 0.001).

To explore the mechanism by which intracellular histidine accumulation promotes *V. splendidus* persister cell formation, we investigated whether histidine modulates *V. splendidus* efflux pump activity via changes PMF (Li et al. 2023). Previous studies have shown that certain amino acids enhance aminoglycoside efflux in bacteria by increasing PMF (Li et al. 2023). A similar phenomenon was observed in *V. splendidus* with tetracycline. Specifically, incubation with exogenous histidine resulted in increased PMF, reduced intracellular tetracycline accumulation and decreased tetracycline‐induced *V. splendidus* persister cell death. Using DiOC_2_(3) (a fluorescent probe for membrane potential), we observed a statistically significant increase in *V. splendidus* membrane potential upon histidine treatment, indicating elevated PMF (Figure 5A,B). Correspondingly, and consistent with the elevated PMF, intracellular tetracycline levels in *V. splendidus* declined with increasing histidine concentration (Figure 5C). Furthermore, the expression of *tolC* was significantly upregulated in histidine‐treated *V. splendidus* (Figure 5D). Transcriptomic analysis also revealed significantly increased FPKM values for other genes involved in ABC transporter pathways (Figure 5E). Together, these results indicate that histidine enhances PMF and efflux pump gene expression in *V. splendidus* , thereby promoting the efflux of tetracycline and ultimately enhancing persister cell formation.

### 3.5 Exogenous Histidine Differentially Modulates *V. splendidus* Persister Cell Tolerance to Different Antibiotics

**FIGURE 6:**
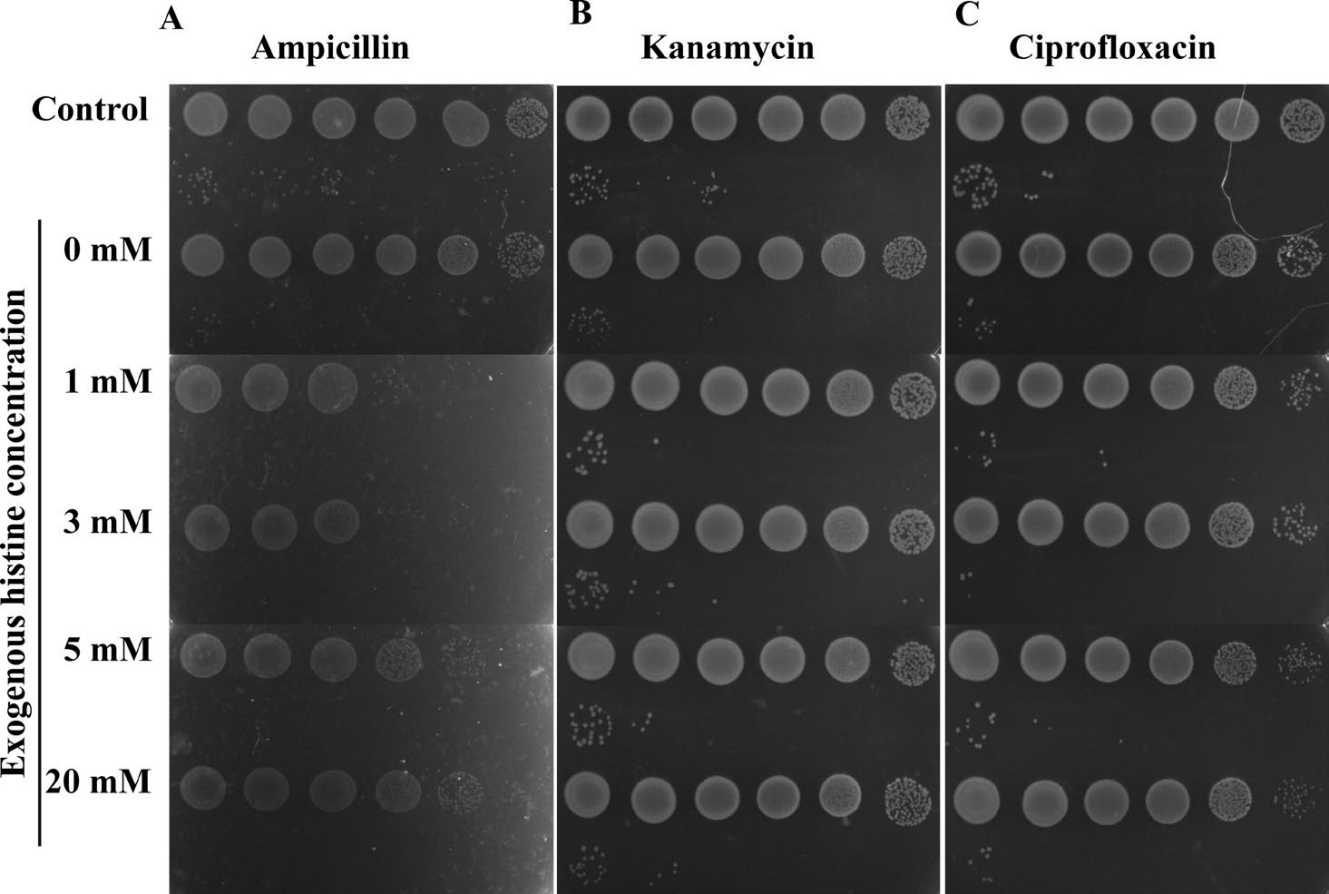
Exogenous histidine differentially modulates *V. splendidus* persister cell tolerance to different antibiotics. Cell survival after exogenous histidine treatment, determined by antibiotics susceptibility measurement. (A) Ampicillin, (B) Kanamycin, (C) Ciprofloxacin.

To determine whether the effect of exogenous histidine on persister cell antibiotic tolerance is specific to tetracycline‐induced *V. splendidus* persister cells, we tested *V. splendidus* persister cell viability under treatment with 10 × MIC concentrations of other antibiotics. *V. splendidus* tetracycline‐induced persister cells were exposed to 10 × MIC concentration of ampicillin, kanamycin, or ciprofloxacin in the presence or absence of exogenous histidine. Interestingly, co‐incubation with ampicillin and exogenous histidine resulted in a significant increase in *V. splendidus* persister cell survival (Figure 6A). In contrast, exogenous histidine exerted no significant effect on *V. splendidus* persister cell tolerance to kanamycin or ciprofloxacin (Figure 6B,C). These results indicate that exogenous histidine specifically enhances *V. splendidus* persister cell tolerance to tetracycline and ampicillin.

## 4 Discussion

The regulation of bacterial metabolic networks is a key mechanism underlying the persistence of bacterial antibiotic tolerance in many bacterial pathogens. For example, the cysteine and methionine metabolic pathways were significantly downregulated in tet(X)‐positive *E. coli* , while exogenous supplementation with L‐methionine (Met) effectively restored the susceptibility of the bacteria to tigecycline (Guo et al. 2025). In addition, the glyoxylate shunt is a metabolic pathway that acts as an alternative to the TCA cycle. It enables bacteria to utilize non‐carbohydrate carbon sources for gluconeogenesis under nutrient‐deficient conditions, thereby sustaining energy homeostasis and cell survival (Cheng et al. 2025). Whether histidine accumulation can enhance the tolerance of *V. splendidus* persister cells was a key question addressed in this study. We performed a global transcriptomic analysis to compare tetracycline‐induced *V. splendidus* persister cells with actively growing *V. splendidus* cells. The transcriptomic FPKM data showed increased expression of most his operon genes in tetracycline‐induced persister cells, except for *hisG* and *hisD*, which were downregulated. HisG and HisD are two key enzymes in the bacterial histidine biosynthesis pathway, which control the first rate‐limiting step and the last two steps of end product formation, respectively. The downregulation of gene expression may be related to the negative regulation when histidine concentration increases. We identified the statistically significant upregulation of genes implicated in the histidine metabolic pathway, indicating that intracellular histidine accumulation confers tetracycline tolerance to *V. splendidus* persister cells—consistent with our ELISA and functional assay results. Accordingly, targeting histidine biosynthesis may serve as a promising strategy for eliminating tetracycline‐induced *V. splendidus* persister cells in aquaculture settings.

In this study, we observed that tetracycline—an antibiotic that inhibits translation by binding the ribosome‐induced a higher level of persistence and deeper dormancy compared to kanamycin, ampicillin and ciprofloxacin (Figure 1E). Analysis of persister cell formation over time revealed a sharp increase during the early exponential phase, which continued into the stationary phase (Figure 1B) and is consistent with the dynamics of persister formation in *E. coli* (Lewis 2007; Keren et al. 2004). A similar trend was observed with ampicillin, kanamycin and ciprofloxacin treatment. Bacteria survival under antibiotics stress is governed not by a single gene or pathway, but by an integrated network of cellular responses (Keren et al. 2011; Kohanski et al. 2010; Dwyer et al. 2014). RNA seq analysis revealed 1.5 times more upregulated than downregulated genes in antibiotic‐induced *V. splendidus* persister cells across three growth stages in this study (Wang et al. 2009). Notably, ribosome metabolism was broadly downregulated, which is consistent with reports in *E. coli* and *M. tuberculosis* (Fang et al. 2025; Guo et al. 2025; Esani et al. 2019). However, among the upregulated genes, the histidine metabolism pathway was notably prominent. Histidine biosynthesis is a model system for understanding the transcriptional, metabolic and autogenous regulation mechanisms (Winkler and Ramos‐Montañez 2009). Histidine is one of the most energetically costly amino acids synthesized by bacteria (Akashi and Gojobori 2002). Previous studies reported that microbes exploit histidine and nitrogen acquisition during host infection (Lonergan et al. 2020). In our study, tetracycline‐induced persister cells exhibited upregulation of histidine biosynthesis genes and elevated intracellular histidine levels. We hypothesized that histidine accumulation contributes to tetracycline tolerance. Exogenous supplementation of histidine validated this hypothesis, as it significantly enhanced persister cell survival under tetracycline treatment. Interestingly, exogenous L‐histidine has also been shown to inhibit bacterial wilt disease in tomato *Solanum lycopersicum* and *Arabidopsis thaliana* without directly affecting the pathogen *Ralstonia solanacearum* , suggesting an indirect antimicrobial role (Seo et al. 2016). In contrast, knockdown or knockout of genes involved in the histidine metabolic pathway in cancer cells led to reduced intracellular histidine levels and diminished sensitivity to methotrexate (Kanarek et al. 2022), indicating context‐dependent roles of histidine metabolism. IGPD, a key enzyme catalysing the sixth step in histidine biosynthesis, is the molecular target of the herbicide 3‐AT, and deletion of the gene encoding IGPD abrogates histidine biosynthesis (Busch et al. 2001; Dietl et al. 2016). In this study, 3‐AT treatment reduced survival of persister cells. These results suggest that 3‐AT is a promising candidate for inhibiting the formation of tetracyclin*e*‐induced *V. splendidus* persister cells.

Efflux of antibiotics via membrane transporters is a major mechanism of both intrinsic and acquired tolerance, particularly in Gram‐negative pathogens such as *Enterobacteriaceae*, *Acinetobacter* and *Pseudomonas*. Efflux pump activity is closely tied to the membrane potential; perturbing membrane permeability and reducing PMF can affect both antibiotic uptake and efflux (Alav et al. 2018; Hajiagha and Kafil 2023). In our experiments, histidine supplementation elevated membrane potential and enhanced expression of *tolC*, the major efflux pump gene, resulting in lower intracellular tetracycline levels. At present, there are increasingly more studies that have focused on the regulation of efflux pumps and membrane potential by amino acid metabolites. In *Lactobacillus reuteri* , the ClC transporter regulates histidine metabolism by altering intracellular pH and membrane potential and significantly increases membrane potential under acidic conditions, confirming a close association between histidine metabolism and membrane potential (Hall et al. 2019). On the contrary, alkaline arginine significantly increases NADH concentration and proton motive force (PMF) in *Salmonella*, enhances the bactericidal efficacy of gentamicin and demonstrates that arginine improves antibiotic susceptibility by boosting membrane potential (Zhu et al. 2023). In addition, L‐lysine potentiates aminoglycosides against *Acinetobacter baumannii* via regulation of PMF and antibiotics uptake (Deng et al. 2020). To summarize, the addition of exogenous amino acids to bacterial cells can affect intracellular metabolic network pathways and may even alter the physiological and biochemical characteristics of bacteria, in particular the efflux pumps (Zeng et al. 2025). Major efflux pumps such as the RND family transporters include those associated with *tolC* and analogous outer membrane factors. In the present study, we observed that histidine accumulation enhanced membrane potential and upregulated the expression of efflux pump‐related genes, thereby reducing intracellular tetracycline levels. These results suggest that histidine metabolism may promote tetracycline tolerance in *V. splendidus* persister cells, at least in part by modulating PMF and boosting the activity of efflux pumps. This regulatory linkage between amino acid metabolism and efflux pump function provides a novel metabolic perspective for understanding tetracycline tolerance in Vibrio.

Our study demonstrated that exogenous histidine enhances the survival of *V. splendidus* persister cells under tetracycline pressure by enhancing intracellular histidine accumulation, increasing PMF and upregulating efflux pump gene expression. Conversely, inhibition of histidine biosynthesis with 3‐AT effectively suppressed *V. splendidus* persister cell formation. These results underscore histidine metabolism as a key regulatory pathway in *V. splendidus* persistence and lay a theoretical foundation for developing novel strategies to prevent and eliminate tetracycline‐induced persister cells in *V. splendidus* in aquaculture.

## Author Contributions

**Xinnan Pan:** investigation, data curation. **Jiayao Chen:** investigation, data curation. **Hong Ding:** investigation. **Yina Shao:** funding acquisition. **Yanan Li:** writing – original draft, visualization, methodology, investigation, formal analysis, data curation, conceptualization.

## Funding

This work was supported by Scientific Research Foundation of Yancheng Teachers University, No. 204070032, No. 212070010; Collaborative Innovation Center of High‐Efficiency and Healthy Marine Aquaculture of Zhejiang at Ningbo University.

## Conflicts of Interest

The authors declare no conflicts of interest.

## Data Availability

The data that support the findings of this study are available on request from the corresponding author. The data are not publicly available due to privacy or ethical restrictions.

## References

[emi470383-bib-0001] Ahangar, M. S. , R. Vyas , N. Nasir , and B. K. Biswal . 2013. “Structures of Native, Substrate‐Bound and Inhibited Forms of *Mycobacterium tuberculosis* Imidazoleglycerol‐Phosphate Dehydratase.” Acta Crystallographica, Section D: Biological Crystallography 69: 2461–2467. 24311587 10.1107/S0907444913022579

[emi470383-bib-0002] Akashi, H. , and T. Gojobori . 2002. “Metabolic Efficiency and Amino Acid Composition in the Proteomes of Escherichia Coli and *Bacillus subtilis* .” Proceedings of the National Academy of Sciences of the United States of America 99: 3695–3700. 11904428 10.1073/pnas.062526999 PMC122586

[emi470383-bib-0003] Alav, I. , J. M. Sutton , and K. M. Rahman . 2018. “Role of Bacterial Efflux Pumps in Biofilm Formation.” Journal of Antimicrobial Chemotherapy 73: 2003–2020. 29506149 10.1093/jac/dky042

[emi470383-bib-0004] Brosnan, M. E. , and J. T. Brosnan . 2020. “Histidine Metabolism and Function.” Journal of Nutrition 150: 2570S–2575S. 33000155 10.1093/jn/nxaa079 PMC7527268

[emi470383-bib-0005] Busch, S. , B. Hoffmann , O. Valerius , K. Starke , K. Düvel , and G. H. Braus . 2001. “Regulation of the *Aspergillus nidulans* hisB Gene by Histidine Starvation.” Current Genetics 38: 314–322. 11270573 10.1007/s002940000171

[emi470383-bib-0006] Cheng, Z. L. , S. Zhang , Z. Wang , et al. 2025. “Pathogen‐Derived Glyoxylate Inhibits Tet2 DNA Dioxygenase to Facilitate Bacterial Persister Formation.” Cell Metabolism 37, no. 5: 1137–1151.e5. 40037360 10.1016/j.cmet.2025.01.019

[emi470383-bib-0007] Deng, W. , T. Fu , Z. Zhang , et al. 2020. “L‐Lysine Potentiates Aminoglycosides Against *Acinetobacter baumannii* via Regulation of Proton Motive Force and Antibiotics Uptake.” Emerging Microbes & Infections 9, no. 1: 639–650. 32192413 10.1080/22221751.2020.1740611 PMC7144275

[emi470383-bib-0008] Derewacz, D. K. , C. R. Goodwin , C. R. McNees , J. A. McLean , and B. O. Bachmann . 2013. “Antimicrobial Drug Resistance Affects Broad Changes in Metabolomic Phenotype in Addition to Secondary Metabolism.” Proceedings of the National Academy of Sciences of the United States of America 110: 2336–2341. 23341601 10.1073/pnas.1218524110 PMC3568320

[emi470383-bib-0009] Dietl, A. M. , J. Amich , S. Leal , et al. 2016. “Histidine Biosynthesis Plays a Crucial Role in Metal Homeostasis and Virulence of *Aspergillus fumigatus* .” Virulence 7: 465–476. 26854126 10.1080/21505594.2016.1146848 PMC4871644

[emi470383-bib-0010] Dwivedy, A. , A. Ashraf , B. Jha , D. Kumar , N. Agarwal , and B. K. Biswal . 2021. “De Novo Histidine Biosynthesis Protects *Mycobacterium tuberculosis* From Host IFN‐γ Mediated Histidine Starvation.” Communications Biology 4, no. 1: 410. 33767335 10.1038/s42003-021-01926-4 PMC7994828

[emi470383-bib-0011] Dwyer, D. J. , P. A. Belenky , J. H. Yang , et al. 2014. “Antibiotics Induce Redox‐Related Physiological Alterations as Part of Their Lethality.” Proceedings of the National Academy of Sciences of the United States of America 111: E2100–E2109. 24803433 10.1073/pnas.1401876111 PMC4034191

[emi470383-bib-0012] Esani, S. , T. Chen , K. P. Leung , and T. A. Van Laar . 2019. “Transcriptome Sequence of Antibiotic‐Treated *Pseudomonas aeruginosa* .” Microbiology Resource Announcements 8: e01367‐18. 30938705 10.1128/MRA.01367-18 PMC6430322

[emi470383-bib-0013] Fang, D. , T. Xu , F. Li , et al. 2025. “Methionine‐Driven Methylation Modification Overcomes Plasmid‐Mediated High‐Level Tigecycline Resistance.” Nature Communications 16, no. 1: 417. 10.1038/s41467-024-55791-w PMC11704046 39762254

[emi470383-bib-0014] Farmer, J. J. 2006. “The Family Vibrionaceae.” In The Prokaryotes, edited by M. Dworkin , S. Falkow , E. Rosenberg , K. H. Schleifer , and E. Stackebrandt . Springer.

[emi470383-bib-0015] Fisher, R. A. , B. Gollan , and S. Helaine . 2017. “Persistent Bacterial Infections and Persister Cells.” Nature Reviews. Microbiology 15: 453–464. 28529326 10.1038/nrmicro.2017.42

[emi470383-bib-0016] Guo, W. , X. Duan , C. Xiao , S. Fu , and L. Shen . 2025. “Identification and Characterization of Alternative Homologs of Histidinol‐Phosphate Phosphatase in *Pseudomonas aeruginosa* .” BMC Microbiology 25, no. 1: 377. 40596842 10.1186/s12866-025-04092-3 PMC12220765

[emi470383-bib-0017] Hajiagha, M. N. , and H. S. Kafil . 2023. “Efflux Pumps and Microbial Biofilm Formation.” Infection, Genetics and Evolution 112: 105459. 10.1016/j.meegid.2023.105459 37271271

[emi470383-bib-0018] Hall, A. E. , M. A. Engevik , N. Oezguen , A. Haag , and J. Versalovic . 2019. “ClC Transporter Activity Modulates Histidine Catabolism in *Lactobacillus reuteri* by Altering Intracellular pH and Membrane Potential.” Microbial Cell Factories 18, no. 1: 212. 31830990 10.1186/s12934-019-1264-0 PMC6909576

[emi470383-bib-0019] Helaine, S. , and E. Kugelberg . 2014. “Bacterial Persisters: Formation, Eradication, and Experimental Systems.” Trends in Microbiology 22: 417–424. 24768561 10.1016/j.tim.2014.03.008

[emi470383-bib-0020] Hinshelwood, S. , and N. G. Stoker . 1992. “Cloning of Mycobacterial Histidine Synthesis Genes by Complementation of a *Mycobacterium smegmatis* Auxotroph.” Molecular Microbiology 6, no. 19: 2887–2895. 1435262 10.1111/j.1365-2958.1992.tb01468.x

[emi470383-bib-0021] Hossain, A. , M. Habibullah‐Al‐Mamun , I. Nagano , S. Masunaga , D. Kitazawa , and H. Matsuda . 2022. “Antibiotics, Antibiotic‐Resistant Bacteria, and Resistance Genes in Aquaculture: Risks, Current Concern, and Future Thinking.” Environmental Science and Pollution Research International 29: 11054–11075. 35028843 10.1007/s11356-021-17825-4

[emi470383-bib-0022] Hossain, A. , M. Raknuzzaman , and M. Tokumura . 2020. “Coronavirus (COVID‐19) Pandemic: Concern About Misuse of Antibiotics.” Journal of Pharmaceutical and Biomedical Analysis 3: 19–23.

[emi470383-bib-0023] Hu, P. , J. Shao , G. Qian , A. S. Adeleye , and T. Hao . 2022. “Removal of Tetracycline by Aerobic Granular Sludge From Marine Aquaculture Wastewater: A Molecular Dynamics Investigation.” Bioresource Technology 355: 127286. 35545206 10.1016/j.biortech.2022.127286

[emi470383-bib-0024] Jiang, G. , Y. Li , Y. Li , W. Zhang , and C. Li . 2022. “Selection of the Amino Acid and Saccharide That Increase the Tetracycline Susceptibility of *Vibrio splendidus* .” Frontiers in Veterinary Science 8: 823332. 35155654 10.3389/fvets.2021.823332 PMC8831740

[emi470383-bib-0025] Kanarek, N. , H. R. Keys , J. R. Cantor , et al. 2022. “Author Correction: Histidine Catabolism Is a Major Determinant of Methotrexate Sensitivity.” Nature 602: E17–E18. 35017686 10.1038/s41586-021-03487-2

[emi470383-bib-0026] Keren, I. , S. Minami , E. Rubin , and K. Lewis . 2011. “Characterization and Transcriptome Analysis of *Mycobacterium tuberculosis* Persisters.” MBio 2: e00100–e00111. 21673191 10.1128/mBio.00100-11 PMC3119538

[emi470383-bib-0027] Keren, I. , D. Shah , A. Spoering , N. Kaldalu , and K. Lewis . 2004. “Specialized Persister Cells and the Mechanism of Multidrug Tolerance in *Escherichia coli* .” Journal of Bacteriology 186: 8172–8180. 15576765 10.1128/JB.186.24.8172-8180.2004 PMC532439

[emi470383-bib-0028] Kohanski, M. A. , D. J. Dwyer , and J. J. Collins . 2010. “How Antibiotics Kill Bacteria: From Targets to Networks.” Nature Reviews. Microbiology 8: 423–435. 20440275 10.1038/nrmicro2333 PMC2896384

[emi470383-bib-0029] Kwan, B. W. , J. A. Valenta , M. J. Benedik , and T. K. Wood . 2013. “Arrested Protein Synthesis Increases Persister‐Like Cell Formation.” Antimicrobial Agents and Chemotherapy 57: 1468–1473. 23295927 10.1128/AAC.02135-12 PMC3591907

[emi470383-bib-0030] Langmead, B. , and S. L. Salzberg . 2012. “Fast Gapped‐Read Alignment With Bowtie 2.” Nature Methods 9: 357–359. 22388286 10.1038/nmeth.1923 PMC3322381

[emi470383-bib-0031] Lewis, K. 2007. “Persister Cells, Dormancy and Infectious Disease.” Nature Reviews. Microbiology 5: 48–56. 17143318 10.1038/nrmicro1557

[emi470383-bib-0032] Li, Y. , T. K. Wood , W. Zhang , and C. Li . 2021. “ *Vibrio splendidus* Persister Cells Induced by Host Coelomic Fluids Show a Similar Phenotype to Antibiotic‐Induced Counterparts.” Environmental Microbiology 23: 5605–5620. 34390618 10.1111/1462-2920.15717

[emi470383-bib-0033] Li, Y. , T. K. Wood , W. Zhang , and C. Li . 2023. “Purine Metabolism Regulates *Vibrio splendidus* Persistence Associated With Protein Aggresome Formation and Intracellular Tetracycline Efflux.” Frontiers in Microbiology 14: 1127018. 37007472 10.3389/fmicb.2023.1127018 PMC10060992

[emi470383-bib-0034] Lim, J. , J. J. Lee , S. K. Lee , S. Kim , S. Y. Eum , and H. Eoh . 2021. “Phosphoenolpyruvate Depletion Mediates Both Growth Arrest and Drug Tolerance of *Mycobacterium tuberculosis* in Hypoxia.” Proceedings of the National Academy of Sciences of the United States of America 118: e2105800118. 34426499 10.1073/pnas.2105800118 PMC8536378

[emi470383-bib-0035] Lonergan, Z. R. , L. D. Palmer , and E. P. Skaar . 2020. “Histidine Utilization Is a Critical Determinant of Acinetobacter Pathogenesis.” Infection and Immunity 88: e00118‐20. 32341119 10.1128/IAI.00118-20 PMC7309604

[emi470383-bib-0036] Moffatt, B. A. , and H. Ashihara . 2002. “Purine and Pyrimidine Nucleotide Synthesis and Metabolism.” Arabidopsis Book 1: e0018. 22303196 10.1199/tab.0018 PMC3243375

[emi470383-bib-0037] Ning, J. , M. Sala , J. Reina , R. Kalagiri , T. Hunter , and B. S. McCullough . 2024. “Histidine Phosphorylation: Protein Kinases and Phosphatases.” International Journal of Molecular Sciences 25, no. 14: 7975. 39063217 10.3390/ijms25147975 PMC11277029

[emi470383-bib-0038] Rydzak, T. , R. A. Groves , R. Zhang , et al. 2022. “Metabolic Preference Assay for Rapid Diagnosis of Bloodstream Infections.” Nature Communications 13: 2332. 10.1038/s41467-022-30048-6 PMC9050716 35484129

[emi470383-bib-0039] Seo, S. , K. Nakaho , S. W. Hong , H. Takahashi , H. Shigemori , and I. Mitsuhara . 2016. “L‐Histidine Induces Resistance in Plants to the Bacterial Pathogen *Ralstonia solanacearum* Partially Through the Activation of Ethylene Signaling.” Plant & Cell Physiology 57: 1932–1942. 27335353 10.1093/pcp/pcw114

[emi470383-bib-0040] Su, Y. B. , B. Peng , H. Li , et al. 2018. “Pyruvate Cycle Increases Aminoglycoside Efficacy and Provides Respiratory Energy in Bacteria.” Proceedings of the National Academy of Sciences of the United States of America 115: E1578–E1587. 29382755 10.1073/pnas.1714645115 PMC5816162

[emi470383-bib-0041] Takemura, A. F. , D. M. Chien , and M. F. Polz . 2014. “Associations and Dynamics of Vibrionaceae in the Environment, From the Genus to the Population Level.” Frontiers in Microbiology 5: 38. 24575082 10.3389/fmicb.2014.00038 PMC3920100

[emi470383-bib-0042] Thompson, F. L. , T. Iida , and J. Swings . 2004. “Biodiversity of Vibrios.” Microbiology and Molecular Biology Reviews 68: 403–431. 15353563 10.1128/MMBR.68.3.403-431.2004 PMC515257

[emi470383-bib-0043] Van den Bergh, B. , M. Fauvart , and J. Michiels . 2017. “Formation, Physiology, Ecology, Evolution and Clinical Importance of Bacterial Persisters.” FEMS Microbiology Reviews 41, no. 3: 219–251. 28333307 10.1093/femsre/fux001

[emi470383-bib-0044] Wang, Z. , M. Gerstein , and M. Snyder . 2009. “RNA‐Seq: A Revolutionary Tool for Transcriptomics.” Nature Reviews. Genetics 10: 57–63. 10.1038/nrg2484 PMC2949280 19015660

[emi470383-bib-0045] Winkler, M. E. , and S. Ramos‐Montañez . 2009. “Biosynthesis of Histidine.” EcoSal Plus 3, no. 6.1.9. 10.1128/ecosalplus.3.6.1.9 PMC4251894 26443768

[emi470383-bib-0046] Wiuff, C. , R. M. Zappala , R. R. Regoes , K. N. Garner , F. Baquero , and B. R. Levin . 2005. “Phenotypic Tolerance: Antibiotic Enrichment of Noninherited Resistance in Bacterial Populations.” Antimicrobial Agents and Chemotherapy 49: 1483–1494. 15793130 10.1128/AAC.49.4.1483-1494.2005 PMC1068602

[emi470383-bib-0047] Zeng, Y. Y. , H. Y. Lin , S. C. Yuan , X. X. Peng , and H. Li . 2025. “Aspartate Potentiates Tobramycin Against Multidrug‐Resistant *Edwardsiella tarda* Through Enhancing Proton Motive Force and Membrane Permeability.” mSystems 10, no. 9: e0079425. 40874620 10.1128/msystems.00794-25 PMC12456000

[emi470383-bib-0049] Zhang, W. , and C. Li . 2021. “Virulence Mechanisms of Vibrios Belonging to the *Splendidus* Clade as Aquaculture Pathogens, From Case Studies and Genome Data.” Reviews in Aquaculture 13: 1–23.

[emi470383-bib-0048] Zhang, W. , R. Yamasaki , S. Song , and T. K. Wood . 2019. “Interkingdom Signal Indole Inhibits *Pseudomonas aeruginosa* Persister Cell Waking.” Journal of Applied Microbiology 127: 1768–1775. 31487414 10.1111/jam.14434

[emi470383-bib-0050] Zhu, C. , Y. Zhou , J. Kang , H. Yang , J. Lin , and B. Fang . 2023. “Alkaline Arginine Promotes the Gentamicin‐Mediated Killing of Drug‐Resistant Salmonella by Increasing NADH Concentration and Proton Motive Force.” Frontiers in Microbiology 14: 1237825. 37795291 10.3389/fmicb.2023.1237825 PMC10546041

